# Development of a Composite Pain Scale in Foals: A Pilot Study

**DOI:** 10.3390/ani12040439

**Published:** 2022-02-11

**Authors:** Aliai Lanci, Beatrice Benedetti, Francesca Freccero, Carolina Castagnetti, Jole Mariella, Johannes P. A. M. van Loon, Barbara Padalino

**Affiliations:** 1 Department of Veterinary Medical Sciences, University of Bologna, Via Tolara di Sora 50, Ozzano dell’Emilia, 40064 Bologna, Italy; aliai.lanci2@unibo.it (A.L.); beatrice.benedetti7@unibo.it (B.B.); carolina.castagnetti@unibo.it (C.C.); jole.mariella2@unibo.it (J.M.); 2 Health Science and Technologies Interdepartmental Center for Industrial Research (HST-ICIR), University of Bologna, Ozzano dell’Emilia, 40064 Bologna, Italy; 3 Department of Clinical Sciences, Equine Sciences, Faculty of Veterinary Medicine, Utrecht University, Yalelaan 112–114, 3584 CM Utrecht, The Netherlands; j.p.a.m.vanloon@uu.nl or; 4 Sporthorse Medical Diagnostic Centre (SMDC), Hooge Wijststraat 7, 5384 RC Heesch, The Netherlands; 5 Department of Agricultural and Food Sciences (DISTAL), University of Bologna, Viale Fanin 46, 40127 Bologna, Italy; barbara.padalino@unibo.it

**Keywords:** pain, welfare, foals, composite scale, behaviour, equine, quality of life

## Abstract

**Simple Summary:**

Recognition of pain is pivotal to its management and essential to enhance horses’ well-being and quality of life. Pain scales are important tools for this purpose. In the last years, various types of pain scales have been developed for adult horses, also considering different sources of pain. So far, only one scale based on facial expressions has been designed for foals. In this study, the first Foal Composite Pain Scale was developed and its application assessed. The scale was piloted on 35 control pain-free foals and 15 foals suffering from different pain-related conditions by multiple observers, and a preliminary analytical process of validation was performed. It was found that, despite some criticalities, the Foal Composite Pain Scale proved to be a valid tool to assess pain and quality of life in foals. Moreover, the criticalities highlighted by this pilot study are essential to refine the scale for future studies.

**Abstract:**

Prompt pain management is crucial in horses; however, tools to assess pain are limited. This study aimed to develop and pilot a composite scale for pain estimation in foals. The “Foal Composite Pain Scale” (FCPS) was developed based on literature and authors’ expertise. The FCPS consisted of 11 facial expressions, 4 behavioural items, and 5 physical items. Thirty-five pain-free foals (Control Group) and 15 foals experiencing pain (Pain Group) were used. Foals were video-recorded at different time points: the Control Group only at inclusion (C), while the Pain Group at inclusion (T1), after an analgesic treatment (T2), and at recovery (T3). Physical items were also recorded at the same time points. Videos were scored twice by five trained observers, blinded to group and time points, to calculate inter- and intra-observer reliability of each scale item. Fleiss’ kappa values ranged from moderate to almost perfect for the majority of the items, while the intraclass correlation coefficient was excellent (ICC = 0.923). The consistency of FCPS was also excellent (Cronbach’s alpha = 0.842). A cut-off ≥ 7 indicated the presence of pain. The Pain Group scores were significantly higher (*p* < 0.001) than the Control Group and decreased over time (T1, T2 > T3; *p* = 0.001). Overall, FCPS seems clinically applicable to quantify pain and improve the judgment of the quality of life in foals, but it needs modifications based on these preliminary findings. Consequently, further studies on a larger sample size are needed to test the feasibility and validity of the refined FCPS.

## 1. Introduction

Freedom from pain, injury, or disease is one of the five freedoms listed in 1965 in the Brambell Report and it is fundamental to ensure the welfare of animals [1]. Significant progress has been made since these basic freedoms were established, particularly in the development of tools to assess pain in animals [2]. Pain can greatly affect quality of life (QoL). Therefore, recognizing and secondarily managing pain is critical in veterinary as well as in human medicine [3]. Pain assessment is difficult because of the lack of a gold standard. In horses, it is particularly challenging since they are non-verbal beings, evolutionally stoic to avoid predation [4,5]. However, it is demonstrated that horses in pain show considerable behavioural changes [4], which can be suitable measures for pain evaluation [6]. Horses tend to be sincere in their behaviour, and if a certain type of behaviour is induced by a painful condition, that will quickly return to normal once the pain is resolved [7]. This pain-related behaviour can be expressed by more specific patterns (e.g., lameness for orthopaedic pain, rolling for colic pain) and possibly also by changes in physiological parameters such as an increase in heart rate [6].

Different methods for the recognition of pain have been developed in horses, such as behavioural indicators, facial expressions, and physical parameters [6]. To date, composite pain scales and facial expression-based pain scales seem to be the most promising tools for pain assessment in horses [8]. In the last 20 years, different pain scales have been described to identify and assess pain in adult horses related to various conditions, such as orthopedic pain [9,10], colic syndrome [11], post-operative period after arthroscopy [12], exploratory celiotomy [13], castration [14], and also laminitis [15], head-related pain [16,17], chronic pain [18] and ocular pain [19].

Pain scales developed for adult horses are not suitable for foals. There are indeed physiological and behavioural differences between foals and mature horses; consequently, discomfort ethograms are unlikely to be fully similar; this poses a further challenge for pain recognition in the young subjects [20,21]. Recognition and monitoring of pain in foals might have even a greater value because pain-related trauma experienced in the neonatal period could persistently alter pain processing, leading to altered pain thresholds and responses as well as aversive behaviours later in life [22]. To the authors’ knowledge, there are no studies describing discomfort ethogram in foals, while only recently, that has been thoroughly described in adult horses [21]. Lately, one study has described the development and application of a pain scale based uniquely on facial expressions in foals [23].

With the hypothesis that a Foal Composite Pain Scale (FCPS) including different behavioural and physiological factors would be a feasible and valid tool for the assessment of pain, the aim of this study was to develop and pilot a composite scale including facial expressions, behavioural, and physical items for pain estimation in foals.

## 2. Materials and Methods

This study was conducted in compliance with the Directive 2010/63/EU of The European Parliament and of the Council of 22 September 2010 on the protection of animals used for scientific purposes [24] and followed the requirements of the International Society for Applied Ethology (ISAE) Ethical Guidelines [25]. Due to the low severity of the treatment and use of commercial animals, specific approval was not needed according to Directive 2010/63/EU. Foals and mares were brought to the Veterinary Facility by their owners for medical or breeding reasons. At hospitalization, written informed consent was gained from the owners prior to taking part in this research.

### 2.1. Development of the Foal Composite Pain Scale

To develop the scale, an extensive literature review on pain-related topics was conducted using studies (i) identified by the authors (*n* = 7) from their knowledge on this topic area; (ii) using “forward citation chasing” entering the keywords “pain”, “animal based indicators”, “composite scales”, “horse behaviour” together with the words “equine” and “foals” in the web search engine “Google Scholar”, “Scopus” and in “PubMed” database; (iii) using backward citation chasing by analysing the bibliography of the references found. Eligible items for the FCPS were reviewed by the same authors gathered for this study, considering their different areas of expertise (equine neonatology, equine internal medicine and equine welfare and behaviour). The selected items were then grouped into three sections: Section I-facial expressions; Section II-behavioural items; Section III-physical items.

### 2.2. Animals

To pilot the FCPS, 50 foals of various breeds, aged from 1 to 88 days, were enrolled in the study (Table 1). They were included and divided into two groups based on the clinical judgment of an experienced clinician in equine medicine (A.L.). In the Control Group, 35/50 healthy pain-free foals were included and in the Pain Group, 15/50 sick foals experiencing pain were included. Both groups were housed at two locations: the Equine Perinatology and Reproduction Unit-EPU-of the University Teaching Hospital (UTH) of Bologna and a local breeding farm connected to the UTH. All foals were housed in individual stalls with their dam, and they were free to nurse and turned out daily if permitted. The Control Group foals remained healthy for the entire duration of the study; the Pain Group foals presented several clinical conditions associated with pain, including abdominal, orthopaedic and surgical sources of pain (i.e., septic arthritis, osteomyelitis, meconium impaction, inguinal hernia, uroperitoneum, post-operative pain). Sick foals were managed individually according to their condition during the study period; an analgesic medication, usually an NSAID (i.e., flunixin meglumine 1.1 mg/Kg IV) was included in the treatment plan. None of them was unable to stand nor unable/prevented to nurse for clinical reasons.

### 2.3. Video Recording

**Figure 1:**
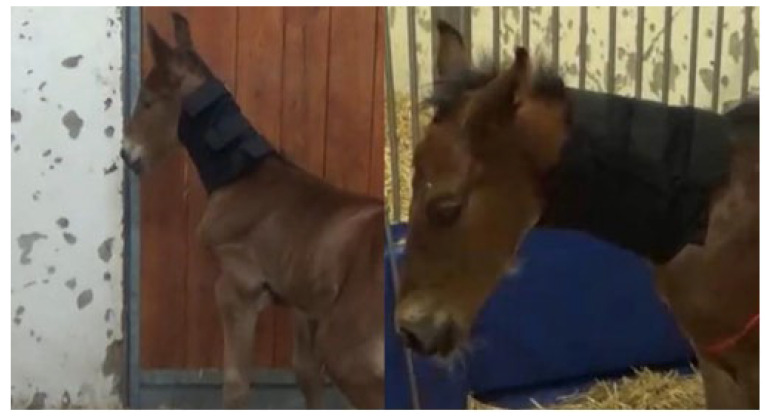
One of the foals used in the study wearing the collar applied to hide the possible presence of an intravenous catheter on the neck during the video recording.

Control Group foals were video-recorded at a single time-point (C), at the inclusion in the study. Pain Group foals were instead recorded three times: at inclusion in the study (T1), two hours after the administration of an analgesic drug (flunixin meglumine 1.1 mg/Kg IV BID, Meglufen^®^, Equality S.r.l., Milan, Italy) (T2) and at recovery (T3). Recovery was decided after a clinical examination by clinicians with experience in neonatology (C.C., J.M., A.L.). At each time point, all foals were recorded while they were in their individual stalls and free to express their behaviours. Videos were taken at different hours of the day/night depending on the time of inclusion in the study or the time of the administration of the analgesic drug. To standardize the video setting, in both groups a black cloth collar was applied to the neck of the foals (Figure 1) before recordings. Once the collar was positioned, several minutes were waited (at least 15 min) until the foal seemed to be comfortable with wearing it and was not showing any discomfort behaviour (e.g., head tossing).

Videos were recorded using a digital video camera recorder (HDR-CX405, Sony, Tokyo, Japan) always by the same operator (B.B), who stood outside the box minimizing interfering with the animals. For each foal, multiple video sequences (*n* = 2–4) from 30 s to 1.5 min in duration were taken over a maximum period of 15–20 min and then combined. During the video recording, some magnifications were made at the level of the head to better visualize the facial expressions of the foal.

Concomitantly to the video recording, while the camera-operator (B.B.) was taking the video, one of the authors (A.L.) gave her score for each item of the scale (that will be referred to as “Clinical judgement”—CJ) by direct observation from outside the box without disturbing the animal. Before performing the clinical examination, respiratory rate was evaluated from outside the stall, watching at the movements of the flank region and counting breaths over 30 s. Immediately after, she entered into the box and performed a clinical examination to score the physical items and fill all the three Sections of the FCPS. Heart rate was evaluated by placing a stethoscope on the left chest at the cardiac area and counting heartbeats over 60 s. The presence of gut sounds was assessed over 30–60 s using a stethoscope placed at the four abdominal quadrants. Reaction to palpation was manually assessed by applying gentle pressure on the potentially painful area (e.g., abdomen, joint, umbilical region) in Pain Group foals and on the abdomen in Control Group foals. Rectal temperature was recorded using a digital thermometer (PIC Solution, PIKDARE S.P.A., Como, Italy).

At the end of the study, a total of 240 video sequences had been recorded and they were combined in 80 individual videos (C = 35, Pain Group = 15*3 time-points = 45). All videos were then reviewed and qualitatively selected by the camera-operator (B.B.) for visual assessment suitability. Out of 80 videos available, 11 were excluded from the analysis due to inadequate quality. The remaining 69 videos were then coded, randomized and pooled together for subsequent evaluations.

### 2.4. Observers and Training

All videos were assessed by 5 observers (A, B, C, D, E) with different degrees of experience in equine medicine and behaviour (1 senior, 3 junior veterinary staff members and 1 veterinary student). Before starting the behavioural analysis on the videos, they had undergone two-day training using detailed descriptions of the items and explanatory videos and images of adult horses and foals (not included in the present study). After the training, a trial with 15 videos of foals (not included in the present study) applying the FCPS was carried out. Once a good level of inter-observer agreement was reached, the observers independently assessed all the video and scored the FCPS, being blinded to foals’ history and clinical condition and time/date of the recording. During the evaluation of the videos, the observers had to assign not applicable (na) if the item was not visible (e.g., low quality of the video, darkness, foal resting during the video and impossibility to score lameness).

Fifteen out of 69 videos, randomly sampled and numerically balanced between groups and time points (C = 6 videos; Pain Group = 3 videos each of T1, T2 and T3), were presented as duplicates in the pool, and scored twice by the observers, in order to test the intra-observer reliability. Therefore, a total of 84 videos relative to the 50 foals at different time points (i.e., for some foals of the Pain group a few times were missing) was assessed by the 5 observers. Each observer scored each item for each foal/time-point once, even if there were multiple video sequences available. A total of 420 FCPS forms were filled (Sections I and II) by the observers (84 videos × 5 observers). All scores assigned to each individual item by the 5 observers were reported on a commercial data sheet (Microsoft Excel^®^, v16.0, Redmond, WA, USA) for subsequent analysis. In the data sheet, a total of 69 FCPS forms filled (Sections I, II and III) by the CJ were also included.

### 2.5. Statistical Analysis

For each of the 5 observers (A, B, C, D, E), scores of individual items were summed to calculate each section score (Section I; Section II) and obtain a Subtotal score (Sections I + II) of the FCPS. Only in the case of the FCPS filled by the clinician (CJ), which included the physical items, a third section score (Section III) was calculated and added to obtain the Total score (I + II + III) of the scale, and descriptive statistics were performed.

The approach used for the validation process is summarised in Table 2. To evaluate the inter-observer and intra-observer agreement (A, B, C, D, E) on the individual items of the FCPS, a Fleiss’ kappa was calculated. Kappa values were interpreted using the following thresholds: 0.21–0.40 = fair, 0.41–0.60 = moderate, 0.61–0.80 = substantial, 0.81–1.00 = almost perfect [26]. The inter-observer agreement was also calculated on the scores of Section I, Section II, and Subtotal using the intraclass correlation coefficient (ICC) test. ICC values for single measures were reported and interpreted as poor (ICC < 0.40), fair (0.40 < ICC < 0.60), good (0.60 < ICC < 0.75), and excellent (ICC > 0.75).

Cronbach’s alpha (coefficient α) was used to evaluate the consistency of Sections I and II of the FCPS using the scores given by the CJ and the 5 observers. The Cronbach’s alpha was also repeatedly assessed by removing each item individually to consider its effect on the consistency of the scale itself.

Spearman correlations were calculated between the FCSP Total score assigned by the CJ and vital parameters (heart rate, respiratory rate, and rectal temperature). Receiver operating characteristic (ROC) analysis was used to estimate the ability of the FCPS applied by the CJ to discriminate between Control and Pain Group foals and to determine the optimal threshold value (cut-off) for discriminating between the two groups.

A Kolmogorov–Smirnov test was used to assess data for normal Gaussian distribution. Since data were not normally distributed, the effect of the Group (Control or Pain) at inclusion (C vs. T1) on the Total score assigned by the CJ was tested with a Mann Whitney U test. The same test was used to test the effect of the Group (Control vs. Pain) on vital items values (rectal temperature, heart rate and respiratory rate). Categorical variables included in Section III (‘Reaction to palpation of the painful area’ and ‘intestinal motility’) were tested using a chi-square test (χ^2^) between Control Group and Pain Group (C vs. T1). For the item ‘reaction to palpation’, the mild and severe reactions (scored 1 and 2, respectively) were considered together. The effect of time (T1 vs. T2 vs. T3) on Total score in the Pain Group was calculated on the score assigned by the CJ using a Friedman ANOVA and the pairwise comparison was performed using Tukey’s test.

Statistical analysis was performed using SPSS software (Statistics version 25 -IBM, SPSS Inc., Chicago, IL, USA). Statistical significance was accepted with *p* < 0.05.

## 3. Results

### 3.1. Development of the FCPS

The FCPS was developed as a descriptive, multifactorial scale and it was divided into three sections (Table 3, Table 4 and Table 5). In each section, items that were assessable by rating frequency and intensity were scored on a three-point scale (0, 1, 2); items assessed only by their presence/absence were scored on a binary scale (0, 2). For the latter, the score assigned to the presence was 2 (instead of 1) in order to give equal weight for each item of the scale to the maximum scores. The total score of the FCPS ranged from 0 to 40.

#### 3.1.1. Section I: Facial Expressions

This section included 11 facial expression items: ‘head’, ‘eyelids’, ‘focus’, ‘corner mouth/lips’, ‘nostrils’, ‘muscle head tone’, ‘yawning’, ‘licking/chewing’, ‘teeth grinding’, ‘moaning’ and ‘ears’ (Table 3). These were all previously included in the EQUUS-FAP FOAL [23], except for the item ‘smacking the lips’ that was changed with ‘licking/chewing’ in the present scale. Each item was carefully described according to the Equine Discomfort Ethogram [21] and according to Dalla Costa et al. [14]. ‘Yawning’, ‘liking/chewing’, ‘teeth grinding’ and ‘moaning’ were scored on a binary scale (0, 2), the others were scored on a three-point scale (0, 1, 2).

#### 3.1.2. Section II: Behavioural Items

This section included four items: ‘signs of abdominal pain’, ‘posture’, ‘appetite’, and ‘lameness’ (Table 4). The ‘signs of abdominal pain’ in foals included the presence of kicking, pawing, rolling, dropping to the ground [27], as described in adult horses by Torcivia and McDonnell [21].

In the present scale, due to a physiological extended recumbency time in foals, the item ‘posture’ was adapted from adult horses [12,28], to include the assessment of either the standing or the recumbent position or both as caught in a video. Abnormalities in posture (e.g., abnormal weight distribution, atypical recumbency, straining to defecate/urinate, generalized muscle tremors) were used according to Torcivia and McDonnell [21]. The item ‘appetite’ was adapted from adult horses [12] using the foal’s typical feeding behaviour [27]. The item ‘lameness’ was included for the assessment of the orthopaedic pain according to a standard lameness scale used in horses [29].

The items of this section were all scored on a three-point scale (0, 1, 2).

#### 3.1.3. Section III: Physical Items

This section included five items: ‘rectal temperature’ [30], ‘heart rate’ [31,32], ‘respiratory rate’ [33], ‘reaction to palpation of the painful area’ [12] and ‘intestinal motility’ [27] (Table 5). Items are scored on a two-point scale (0, 2), except for ‘reaction to palpation of the painful area’ that is scored by intensity (0, 1, 2).

### 3.2. FCPS Analysis

During the analysis, 211 out of 6300 (3.35%) values were ‘not available’ (na) since it was not possible to score them from the videos. In particular, the most frequently na data were the following: ‘appetite’ (142/211, 67%), ‘lameness’ (46/211, 22%), ‘corner mouth/lips’ (17/211, 8%), ‘licking/chewing’ (6/211, 3%).

Descriptive statistics of the partials and Total Scores are reported in Table 6. The minimum value of the Total score was 0 in a control foal, while the maximum value was 24/40 in a foal in pain due to an intestinal volvulus.

Descriptive statistics of categorical variables included in the Section III are reported in Table 7.

The Fleiss’ kappa values (k) for inter-observer reliability of each item of Sections I and II calculated among the five observers are reported in Table S1 (Supplementary Materials). The item ‘teeth grinding’ (k range = 0.651–1.000) resulted for the majority of the observers in the category almost perfect reliability (k = 0.81–1.00). The items ‘focus’ (k range = 0.471–0.904), ‘yawning’ (k range = 0.485–1.000), ‘licking/chewing’ (k range = 0.403–0.970), ‘moaning’ (k range = 0.385–1.000), ‘ears’ (k range = 0.484–0.773), ‘signs of abdominal pain’ (k range = 0.645–0.831), ‘posture’ (k range = 0.645–0.831), and ‘lameness’ (k range = 0.595–0.823) resulted for the majority of the observers in the category substantial reliability (k = 0.61–0.80). The items ‘head’ (k range = 0.452–0.672), ‘eyelids’ (k range = 0.349–0.689), ‘corner mouth/lips’ (k range = 0.304–0.668), ‘nostrils’ (k range = 0.235–0.699), and ‘muscle head tone’ (k range = 0.359–0.806) resulted for the majority of the observers in the category moderate reliability (k = 0.41–0.60). For the item ‘appetite’, due to the presence of a high value of ‘na’, it was often not possible to perform the analysis.

Intra-observer reliability values for each item of Sections I and II are reported in Table S2 (Supplementary Materials). The items ‘licking/chewing’ (k range = 0.444–1.000), ‘teeth grinding’ (k range = 0.576–1.000), ‘yawning’ (k range = 0.634–1.000), ‘signs of abdominal pain’ (k range = 0.474–1.000), and ‘lameness’ (k range = 0.500–1.000) resulted for the majority of the observers in the category almost perfect reliability (k = 0.81–1.00). The items ‘head’ (k range = 0.667–0.896), ‘eyelids’ (k range = 0.688–0.877), ‘focus’ (k range = 0.468–1.000), ‘nostrils’ (k range = 0.586–0.898), ‘ears’ (k range = 0.384–0.884), and ‘posture’(k range = 0.497–0.795) resulted for the majority of the observers in the category substantial reliability (k = 0.61–0.80). The items ‘corner mouth/lips’ (k range = 0.264–1.000) and ‘muscle head tone’ (k range = 0.286–1.000) resulted for the majority of the observers in the category moderate reliability (k = 0.41–0.60). Regarding the items ‘appetite’ and ‘moaning’, due to the presence of a high value of ‘na’, it was often not possible to perform the analysis.

Inter-observer reliability values on the scores of Section I and Section II and on Subtotal score are reported in Table S3 (Supplementary Materials). The ICC value was always excellent: Section I ICC = 0.894 (Confidence Interval-CI: 0.855–0.927); Section II ICC = 0.884 (CI: 0.842–0.920); Subtotal score ICC = 0.923 (CI:0.893–0.947).

Internal consistency and item-to total correlation results are reported in Table 8. Cronbach’s alpha (0.842) indicated a good internal consistency of the FCPS (Sections I and II scored by CJ and the 5 observers). When items ‘muscle head tone’, ‘yawning’, ‘liking/chewing’, and ‘appetite’ were removed, a mild improvement in the overall Cronbach’s alpha values occurred, indicating that those items did not contribute significantly to the FCPS.

Spearman correlations between the Total score and rectal temperature (r = 0.311, *p* < 0.05), heart rate (r = 0.560, *p* < 0.01), and respiratory rate (r = 0.368, *p* < 0.01) were from low to moderate.

ROC analysis showed that the optimal Total score cut-off value to predict the presence of pain was ≥7 (Sensitivity—Se = 100%; Specificity—Sp = 100%). When considering only the Section I, the cut-off value was ≥5 (Se = 93%; Sp = 100%) (Table S4).

**Figure 2:**
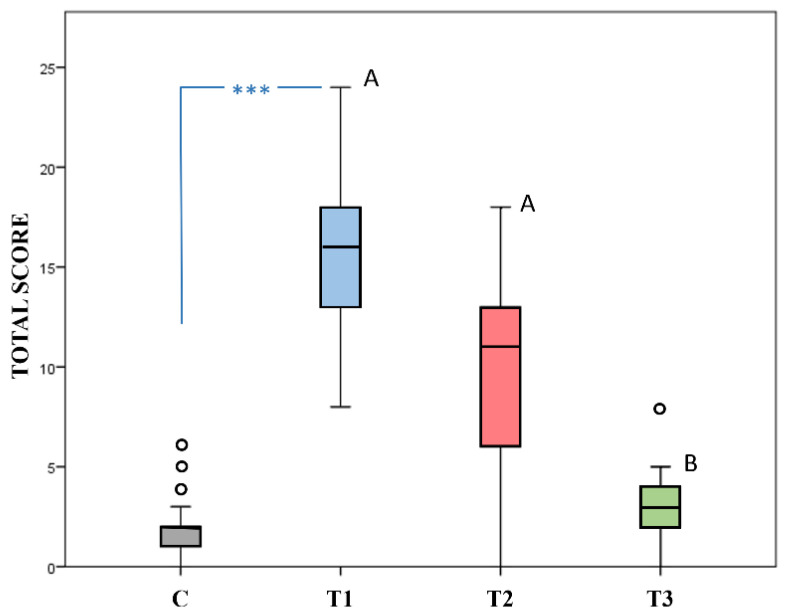
FCPS Total scores recorded in 35 healthy pain-free foals (Control Group = C) and in 15 sick foals experiencing pain at three different time points (T1: at inclusion in the study; T2: after anti-inflammatory drugs; T3: at recovery). The presence of *** indicates a significant difference between groups at inclusion (C vs. T1, Mann Whitney U test *p* < 0.001). Different superscript letters (A, B) indicate a significant difference (*p* < 0.001) between time points (Tukey’s test, T3 vs. T2, T1). Outliers are shown as dots °.

The effect of the Group at inclusion (C vs. T1) was significant for the Total score (Mann Whitney U test, *p* = 0.001), that was higher in the Pain Group with respect to the Control Group (Figure 2).

In the Pain Group, the effect of time on the Total score was significant (Friedman ANOVA, *p* = 0.001). The total score at T3 was significantly lower than the score recorded at T2 (*p* = 0.004) and at T1 (*p* < 0.001); there was no significant difference between T1 and T2 (*p* > 0.05) (Figure 2).

**Figure 3:**
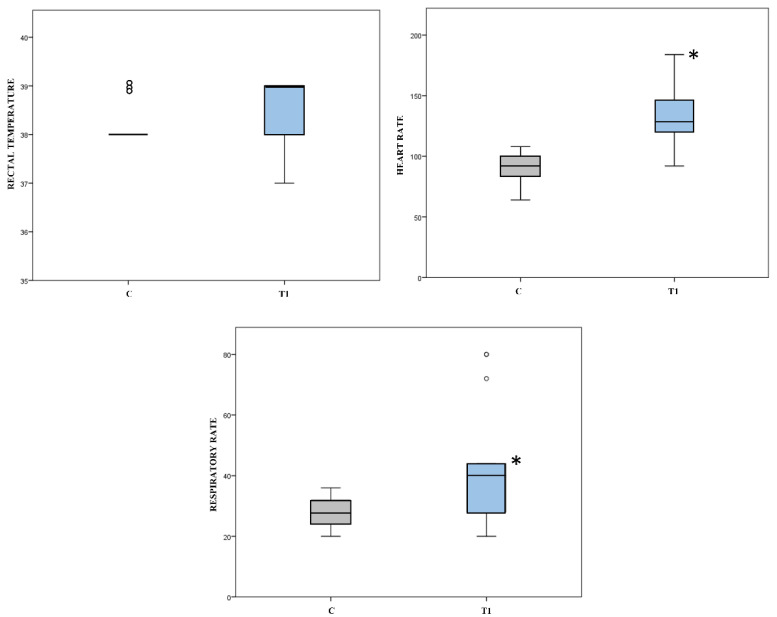
Values of rectal temperature (°C), heart rate (bpm) and respiratory rate (bpm) recorded in 35 healthy pain-free foals (Control Group = C) and in 15 sick foals experiencing pain (T1) at the time of inclusion in the study. Asterisks * indicate a significant difference between groups at inclusion (C vs. T1, Mann Whitney U Test *p* < 0.05). Outliers are shown as dots °.

The effect of the Group at inclusion (C vs. T1) on the rectal temperature values was not significant, while it was significant for heart rate (*p* = 0.001) and respiratory rate (*p* = 0.005). (Figure 3). There was no difference in categorical variables of Section III (‘reaction to palpation of the painful area’ and ‘intestinal motility’) between groups at inclusion (C vs. T1).

## 4. Discussion

This study described the development and the preliminary application of the first composite pain scale in foals (FCPS). Results confirmed the hypothesis, as FCPS showed an excellent consistency and a good intra- and inter-observer agreement for the majority of the items. Therefore, it seems that the FCPS could be a feasible and valid tool to assess pain in foals. Notwithstanding the present preliminary study provides ground, the scale needs further validation. Thus, the results obtained from this pilot study are crucial to refine the FCPS. Since recognizing pain is the first step for its adequate management [3] and it is crucial to improve welfare, these results contribute to improve the QoL of foals.

Since a pain-related ethogram in foals had not been described, in the process of developing the FCPS, the work of the research group was essential to the revision and inclusion of possible meaningful items. While the facial expressions (Section I) of the FCPS were almost fully extracted from the published EQUUS-FAP FOAL scale [23], the item ‘smacking with lips’ was changed with a ‘licking/chewing’ as a result of a consensus reached among the authors. ‘Smacking with lips’ represents a submissive rather than pain-related behaviour [34], since it is often observed in healthy foals in the presence of humans and dominant/older conspecific. In adult horses, ‘smacking with lips’ was distinguished from ‘licking/chewing’ by involving only movement of the lips, whereas ‘licking/chewing’ involved action of the tongue and movement of the mandible, as in a chewing motion [35]. Behavioural items (Section II) were substantially adapted from composite pain scales published in adults [12,28], in light of the intrinsic peculiarities of the foal. Based on clinical experience and debate among the authors, in the present study, the item ‘standing posture’ and ‘recumbency’ were combined in ‘standing and recumbency posture’. If two distinct items were used, the FCPS would reach a higher score even in healthy control foals, since young foals normally spend more and longer time than adult horses in recumbency [20]; furthermore, during recumbency, foals can express subtle but significant signs of discomfort, such as generalized muscle tremors and restlessness [36]. Another typical behaviour of the foal is frequent nursing, which can be reduced or absent, or the foal can stimulate the udder without actually drinking as a sign of discomfort [27]. Section III was conceived to include vital and some other physical items potentially related to pain that can be assessed during the clinical examination. The item ‘reaction to palpation of the painful area’ was included in the present study based on previous findings that suggested it as a valid tool in adult horses [12,28]. In the present study, it did not seem discriminative between control foals and foals with pain. One of the reasons may be that foals had not been similarly handled by humans from birth and there might be a confounding effect of an excessive/inappropriate response to handling or touch [37]. This may be even enhanced by the heterogeneity of breeds included in this study, and potentially by the body region tested. Moreover, ‘intestinal motility’ was found not significant and this partially confirms the results found in the adult horse [12,38,39]. Differently from the above mentioned physical items, vital parameters (heart rate, respiratory rate and rectal temperature) are objective means of assessment. Similarly to what has been done in other pain scales in adult horses [9,12,13,19,28,40,41,42,43], their relationship with the subjective visual evaluation was tested. In the present study, albeit vital items resulted significantly correlated with pain (i.e., Total score), the correlation with ‘rectal temperature’ and ‘respiratory rate’ was low. In fact, in the presence of pain, the rectal temperature often remained within the normal ranges, as expected [12,43]. ‘Respiratory rate’ is easily affected by external factors, such as environmental conditions, and has been described as both stress-related and exploratory behaviour [12,39,42,43,44]. These factors are expected to be even enhanced in foals, despite the attempt to reduce the operator influence by standing outside of the stall. Regarding ‘heart rate’, the correlation with FCPS score was higher, indicating that heart rate could proportionally increase with the pain intensity. However, it is also true that pain may be present without such an increase in heart rate [4]. In adult horses, that correlation has shown controversial results [12,13,40,41,42,43,45]. Other factors (such as temperament, drug administration, and hypovolemia) can affect heart rate, confounding any association between tachycardia and pain [46,47].

In the present study, once the FCPS was developed, intense training of the observers on behaviours’ definition and scoring allowed to reduce some subjectivity in their interpretation and achieve a sustainable agreement among them. Differently from van Loon and van Dierendonck [28], the training in the present study included images and videos of adult horses and foals with various conditions associated with pain, allowing the observers to familiarize themselves with different types and degrees of manifestations of pain-related behaviours. It is the authors’ opinion that the training may render partly the reason of the optimal performance on the reliability of the overall FCPS (Sections and Subtotal), similar to what has been reported with a facial pain scale in foals [23] and some scales used in adult horses [5,12,14,47].

The inter-observer agreement was more variably performing when the individual items of the FCPS sections were tested to insight their potential usefulness and possible weakness. Indeed, when looking at its trend across the observers, the inter-observer reliability was classified as almost perfect on a few items, suggesting they are repeatable, while the majority of items reached a substantial agreement, and some items were classified as moderate. In details, among the facial expressions, the item ‘nostrils’ showed the lowest inter-observer agreement. This was already observed by van Loon et al. [23], probably because flared nostrils observed in healthy pain-free foals could be an explorative behaviour so that it might not be uniformly interpreted [48,49]. With this regard, it is pivotal to provide sufficient time for the assessment of this item, because differently from the sniffing (explorative behaviour), nostrils appear more open for a longer period in case of pain [14]. On the opposite, the items showing the higher inter-observer reliability were ‘focus’, ‘teeth grinding’, ‘moaning’, and ‘ears’; this is probably because they are the easiest and most immediate to recognize by watching/listening to a video. Items such as ‘head’, ‘eyelids’, ‘muscle head tone’, and ‘corner mouth/lips’ have not reached the almost perfect inter-observer reliability; this was likely due to suboptimal video quality, especially when recorded at night, and when the foal had a dark coat, as previously suggested [14]. Regarding Section II, the item ‘appetite’ was difficult to assess by a short single video as it would require a longer time. As an alternative, testing for appetite as it is critical in adult horses [12,38] would be hardly applicable in younger foals, which should be allowed to freely nurse whenever clinically advisable. In contrast, similar to Bussieres et al. [12], the item ‘posture’ showed an excellent inter-observer agreement. As previously mentioned, albeit there are some weak items in both Sections, the inter-observer agreement performed optimally overall. This suggests that the observers were able to score similarly and thus to consistently quantify pain, similar to what was reported for a facial pain scale in foals [23] and multiple scales in adult horses [5,12,14,47].

Concerning the intra-observer agreement for the individual items, it resulted good as a trend, but not as optimal as advisable. We attempted to improve the blinded and randomized fashion of the assessment, in that the observers were not aware of watching the same videos twice. For the same purpose, in a recent study [23], videos were evaluated a second time after two weeks from the first evaluation. Previously, another research group, [50] only tested the intra-observer reliability with one duplicate video. Another potential factor is that in the present study, the observers had to focus their attention simultaneously on many (i.e., 15) different items during each video evaluation. Furthermore, multiple sequences were combined for each foal, so that the attention of the observers might have been caught by different timeframes or elements while scoring an individual item. These factors might have affected the level of inter- and intra- agreement. For the intra-observer reliability, another reason could be that only 15 videos were used, and these might not be enough to reach an optimal agreement. In light of these considerations, future studies need to be repeated using a single video of relatively short and uniform duration for each foal, scoring fewer items, and increasing the number of the videos in duplicate.

The FCPS demonstrated an excellent consistency (Cronbach’s alpha = 0.842), similar to what was shown for the one based on facial expressions by van Loon et al. [23]. However, eliminating the ‘muscle head tone’, ‘yawning’, ‘licking/chewing’, and ‘appetite’ as well as ‘head position’ and ‘ear movements’ items would improve the overall Cronbach’s alpha. As previously observed by van Loon et al. [23], the items ‘muscle head tone’, ‘moaning’, ‘yawning,’ and ‘teeth grinding’ were rarely seen in both healthy controls and foals with pain. For this reason, they were considered poorly discriminative of pain. In particular, yawning was found less consistently. It is described in both the physiological and the pain-related ethograms: in fact, when it is manifested frequently it is associated with discomfort in adult horses [21]. The item ‘licking/chewing’ may have resulted poorly correlated with the scale because it can be shown even in case of boredom/discomfort/stress [21]. Although the videos were acquired with the operator outside the box and interfering as little as possible with foals, in some cases, it was difficult to hide his/her presence. This may have led to the manifestation of some stress behaviours, including licking/chewing lips. Interestingly, it is reported that, in adult horses, most of the behaviours related to discomfort are interrupted by the presence of a person, and then resumed once he/she leaves [21]. Certainly, the best way to obtain videos of undisturbed foals would be to install and activate remote cameras.

The FCPS was able to predict the presence of pain with high sensitivity and specificity. When only the facial expressions were considered, the threshold was very similar to the one (>3) obtained by van Loon et al. [23], further confirming the validity of this section of the scale. It is worth noting that there were no foals assigned with a very high FCPS score in our study. This could be because some of the items currently included do not correlate with pain. Another possible reason is that the assessment is based on short videos and not all the pain-related behaviours could have been manifested in such a short time even in foals with potentially severe pain conditions. However, in further studies, when FCPS will include only the most representative items and will be evaluated on a standardized time window, the cut off values might change. The validity of FCPS was also proven by the fact that the scale was able to discriminate the Group at the time of inclusion, having foals in pain higher scores than controls, as expected [23], and by the lowering of the scores at T3 with the resolution of the clinical condition causing pain. Based on the present findings, the FCPS would benefit from modifications removing some items as well as differential weighting of the items in their contribution to the Total score. A shorter scale would be easier and faster and more practical [51]. Items with poor correlation with pain or redundant (muscle head tone, licking/chewing, yawning, appetite and lameness) could be removed. The same consideration can be done on the items that were rarely seen both in healthy and painful foals (e.g., moaning): removing them will not impact the performance of the scale. Moreover, the items of Section III, which have been shown to have poor correlation with the Total score, will not be considered in further studies. Systematic reviews of pain assessment in humans have shown that physiological parameters, although objective and quantifiable do not reliably represent pain. This is true in infant pain scales [52], in children pain scales [53], as well as scales for demented adults [54]. Furthermore, reducing or avoiding a physical interaction with animals would increase the safety of the operator, as it is advisable for assessing pain in large animals [14], especially in view of a potential application of pain scales by less experienced operators. Finally, it is worth highlighting that for this study all the observers must have undertaken training, which was essential to test the reproducibility of the items. However, in further studies, once the items are refined and the description of each item improved, the feasibility of the FCPS will be tested employing non-trained observers.

Our results need to be interpreted with caution, because this preliminary study has several limitations. First of all, the scale from 0 to 40 was assumed to be linear on the whole, even if it cannot be assumed to be absolutely linear. This is a problem in common with all other equine composite pain scales. The FCPS should have been analyzed with ordinal regression analysis, but considering the high number of items and scores, and the low number of foals included, this was not possible. Moreover, the inability to make the observers completely blind to the foals’ clinical conditions, as was previously noted [23]. Albeit our effort to homogenize the setting, e.g., with a neck collar, some foals did show other features that suggested the presence of a pathological condition (e.g., hair clipping of some areas, suture material, urinary catheter). This could have partly influenced the observers’ judgment. However, because observers were blinded to time points, these factors should have had limited influence on scoring the Pain Group. Other limitations were represented by the small sample size of the enrolled foals and by differences in signalment (age, gender and breed) between the two groups, which we were not able to balance since this was an opportunistic study. Another limitation is the heterogeneity of the places, even though foals were always recorded in their stalls, and also of the time (day vs. night), where different light conditions have likely affected video quality. Last, the pain group was composed by foals with different sources of pain.

Notwithstanding those limitations, this is the first pilot study on a composite pain scale for foals and set the basis for the refinement of the composite pain scale and study protocols that will be applied in future studies. Moreover, the present results are novel and useful to improve the judgment of the QoL in foals. The interest in animal welfare and QoL has considerably grown, and its assessment can provide a firm basis for the implementation of animal welfare standards. An essential aspect of QoL is the health implying that animals should not be affected by any disease [55,56]. Indeed, diseases can cause unpleasant sensations, including pain, which can greatly affect QoL. Equine practitioners should perform QoL assessment regularly and with competence [57]. Nevertheless, the tools that veterinarians have to assess pain objectively are limited, especially for less-experienced members of the professional community. Overall, the developed FCPS could help not only the clinician who is not familiar with the foals’ pain-related behaviour, but also the stable technician who has no clinical background. Our results are consequently useful to educate all foal-related people and to enhance foals’ QoL.

## 5. Conclusions

This preliminary study presented and piloted the first Composite Scale for the estimation of pain in foals. The FCPS seemed to be a feasible and valid tool with a good inter- and intra-observer reliability of the majority of the items when applied by adequately trained observers. The scale had also a good consistency, but further studies are needed to confirm our results and implement the FCPS in light of criticisms and weaknesses highlighted in this preliminary study. Our study is therefore the first step towards improving the foal’s quality of life. This study provides indeed a tool that, with further refinement, may be valid to assess pain in foals in order to ensure its prompt management.

## Figures and Tables

**Table 1:** Demographic details of the 50 foals used in the study. Categorical data are expressed as absolute values and percentage (%) while foals’ age is expressed as mean ± standard deviation (min–max values).

Patients’ Data	Control Group (*n* = 35)	Pain Group (*n* = 15)
Age (days)	33 ± 26 (1–88)	13 ± 12 (1–47)
Males	17/35 (49%)	12/15 (80%)
Females	18/35 (51%)	3/15 (20%)
Breeds	31/35 (88%) Stb 1/35 (3%) Arab 1/35 (3%) QH 2/35 (6%) Oth	6/15 (40%) Stb 2/15 (13%) Arab 3/15 (20%) QH 4/15 (27%) Oth

Stb: Standard bred; Arab: Arabian; QH: Quarter Horse; Oth: Other breeds (Paint Horse, Bardigiano, Italian Heavy Draft Horse and Holstein).

**Table 2:** Statistical methods employed in the validation process of the FCPS.

Type of Analysis	Description	Statistical Test
Inter-observer reliability	Agreement among the five blinded observers	Fleiss’ kappa and ICC for single measures
Intra-observer reliability	Agreement between scores assigned by the same observer to videos viewed twice	Fleiss’ kappa
Internal consistency	Agreement between individual items of the scale and value of Cronbach’s alpha if each item is deleted	Cronbach’s alpha
Construct validity	Degree of correlation of the scale with rectal temperature, heart rate, and respiratory rate theoretically related to pain	Spearman’s rank coefficient of correlation
Construct validity	Cut-off value of the scale to discriminate between pain and no-pain	Receiver operating characteristic (ROC) analysis
Construct validity (Group effect)	Score variation between Control Group and Pain Group	Mann Whitney U test
Construct validity (Time effect in the Pain Group)	Score variation when pain decreases (T1-T2-T3)	Friedman ANOVA test and Tukey’s test

**Table 3:** Score sheet of the Section I of FCPS: Facial expressions. This Section was extracted and modified from previous studies: Dalla Costa et al., 2014; van Loon et al., 2020; Torcivia and McDonnell, 2021 [14,21,23].

Facial Expression Items	Categories	Score
Head	Normal head movement	0
	Less movement/increased movement (low head carriage/head shaking)	1
	No movement/strongly increased movement (low head carriage/head shaking)	2
Eyelids *	Opened, sclera can be seen in case of eye/head movement	0
	More opened eyes/tightening of eyelids	1
	Obviously more opened eyes/obvious tightening of eyelids	2
Focus	Focused on environment (interacts with the surrounding)	0
	Less focused on environment (sometimes depressed and sometimes alert)	1
	Not focused on environment (always depressed)	2
Nostrils *	Relaxed	0
	A bit more opened, sometimes relaxed sometimes flared	1
	Obviously more opened, nostril always flaring and possibly audible breathing	2
Corner mouth/lips *	Relaxed	0
	Lifted slightly	1
	Obviously lifted	2
Muscle head tone *	No fasciculations	0
	Mild fasciculations	1
	Obvious fasciculations	2
Yawning	Not seen	0
	Seen, once or more times	2
Licking/chewing	Both behaviours are not seen	0
	Seen, one or both behaviours, once or more times	2
Teeth grinding	Not heard	0
	Heard, one or more times	2
Moaning	Not heard	0
	Heard, once or more times	2
Ears	Position: orientation towards sound/clear response with both ears or ear closest to source	0
	Reduced movements and delayed/reduced response to sounds	1
	No movement, held backwards and no response to sounds	2
Score		…/22

The presence of * indicates that when it is not possible to score the item (e.g., it is too dark), not applicable (na) should be given.

**Table 4:** Score sheet of Section II of FCPS: Behavioural items. This Section was developed and modified from other studies: AAEP Guide for veterinary service and judging of equestrian events, Bernard, 2004; Bussieres et al., 2008; van Loon and van Dierendonck, 2015; Torcivia and McDonnell, 2021 [12,21,27,28,29].

Behavioural Items	Categories	Score *
Signs of abdominal pain	No kicking, quietly standing without pawing/rolling/dropping to the ground	0
	Occasionally (1–2 times) kicking/rolling/dropping to the ground/pawing	1
	Excessive (>2 times) kicking/rolling/dropping to the ground/pawing	2
Posture (Standing and recumbency)	Stands quietly, normal walk; normal recumbency, no weight shift Occasional weight-shift (1–2 times), slight muscle tremors, but normal recumbency	0
	Non-weight bearing, abnormal weight distribution, analgesic posture, attempts to urinate or defecate, prostration, generalized muscle tremors, prolonged or restless recumbency	1
	Non-weight bearing, abnormal weight distribution, analgesic posture, attempts to urinate or defecate, prostration, generalized muscle tremors, prolonged or restless recumbency	2
Appetite *	Feeds normally to the udder, interested in milk	0
	Shows interest in milk, but drinks very little or stimulates the udder but does not drink	1
	Not interest	2
Lameness *	Not seen	0
	Moderate (I–II degree)	1
	Severe (III–IV degree)	2
Score		…/8

The presence of * indicates that when it is not possible to score the item (e.g., the foal was resting during all video and it was impossible to assess the lameness), not applicable (na) should be given.

**Table 5:** Score sheet of Section III of FCPS: Physical items. This section was developed and adapted from Bussieres et al., 2008; Bernard, 2004 [12,27].

Physical Items	Categories	Score
Rectal temperature	In range (37.2–39.2 °C) [30]	0
	Out of range	2
Heart rate	In range (0–30 days 80–100 bpm/min-1–6 months 45–89 bpm/min) [31,32]	0
	Out of range (tachycardia > 115 bpm/min)	2
Respiratory rate	In range (20–40 breaths/min) [33]	0
	Out of range (dyspnea or tachypnea > 56 breaths/min)	2
Reaction to palpation of the painful area	No reaction to palpation	0
	Mild reaction to palpation	1
	Severe reaction to palpation	2
Intestinal motility	Normal motility	0
	Not normal motility (increase/decrease/absent)	2
Score		…/10

**Table 6:** Descriptive statistics of partial and Total scores by group and time points (Control Group at inclusion, C; Pain Group at different time points, T1 = at the inclusion in the study; T2 = after the administration of an analgesic drug; T3 = at recovery). Data are expressed as median and interquartile range (IQR).

Time Points	Section I Score (0–22)	Section II Score (0–8)	Section III Score (0–10)	Subtotal Score (I + II; 0–30)	Total Score (I + II + III; 0–40)
C	1 (0–2)	0	1 (0–1)	1 (0–2)	2 (1–2)
T1	7 (6–9)	3 (3–5)	4 (4–7)	11 (9–13)	16 (13–18)
T2	6 (3–8)	3 (1–4)	2 (1–3)	10 (5–10)	11 (6–13)
T3	1 (1–1)	0	0	1 (1–2)	3 (2–4)

**Table 7:** Frequency distribution of scores obtained for categorical variables of the Section III (‘reaction to palpation of the painful area’ and ‘intestinal motility’) by group and time points (Control Group at inclusion, C; Pain Group at different time points, T1 = at the inclusion in the study; T2 = after the administration of an analgesic drug; T3 = at recovery). Data are expressed as *n* (%).

Items	Scores	C	T1	T2	T3
Reaction to palpation of the painful area	0	19/35 (54%)	4/15 (27%)	6/13 (46%)	5/6 (83%)
	1	15/35 (43%)	4/15 (27%)	4/13 (31%)	1/6 (17%)
	2	1/35 (3%)	7/15 (46%)	3/13 (23%)	0/6 (0%)
Intestinal motility	0	33/35 (94%)	12/15 (80%)	13/13 (100%)	6/6 (100%)
	2	2/35 (6%)	3/15 (20%)	0/13 (0%)	0/6 (0%)

**Table 8:** Internal consistency and item–total correlation of the Sections I and II of the FCPS using the scores given by the clinician (CJ) and the 5 observers. The Cronbach’s alpha was also repeatedly assessed by removing each item individually to consider its effect on the consistency of the scale itself.

Items	Corrected Item–Total Correlation	Cronbach’s Alpha if the Item Is Removed
Head	0.623	0.823
Eyelids	0.566	0.828
Focus	0.489	0.835
Corner mouth/lips	0.623	0.827
Nostrils	0.545	0.828
Muscle head tone	0.187	0.844
Yawning	0.119	0.845
Liking/chewing	0.439	0.854
Teeth grinding	0.431	0.835
Moaning	0.383	0.838
Ears	0.637	0.823
Signs of abdominal pain	0.390	0.838
Posture	0.839	0.803
Appetite	0.146	0.845
Lameness	0.737	0.813

## Data Availability

Individual raw data collected are available on request from the corresponding author.

## Supplementary Materials

The following supporting information can be downloaded at: https://zenodo.org/record/6252384#.Yhb2JpYRVPZ, Table S1: Interobserver reliability on individual items (Fleiss’ kappa), Table S2: Intra-observer Reliability (Fleiss’ kappa); Table S3: Inter-observer reliability on the Section I and Section II and on Subtotal score (ICC); Table S4: Area under the ROC curves and their confidence intervals. Video S1/S2/S3: exemplificative videos of FCPS scoring of a foal in the Pain Group at T1. The items Yawning, Teeth grinding, Muscle head tone and Moaning were scored 0 points in all the three videos. Video S1: Head = score 1; Ears = score 1; Eyelids = score 1; Licking/chewing = score 2; Signs of abdominal pain = score 2. Video S2: Appetite = score 1; Standing/recumbency posture = score 2. Video S3: Focus= score 0; Head = score 1; Ears = score 1; Corner mouth/lips = score 1; Nostrils = score 2; Lameness = score 2.

## References

[1] Brambell F.W.R. (1965). Report of the Technical Committee to Enquire into the Welfare of Animals Kept under Intensive Livestock Hus-Bandry Systems.

[2] Mullan S. (2015). Assessment of quality of life in veterinary practice. Developing tools for companion animal carers and veterinarians. J. Vet. Med. Res..

[3] Max M.B., Donovan M., Miaskowski C.A., Ward S.E., Gordon D., Bookbinder M., American Pain Society Quality of Care Committee (1995). Quality improvement guidelines for the treatment of acute pain and cancer pain. JAMA.

[4] Taylor P.M., Pascoe P.J., Mama K.R. (2002). Diagnosing and treating pain in the horse: Where are we today?. Vet. Clin. North. Am. Equine Pract..

[5] Van Loon J.P., Back W., Hellebrekers L.J., van Weeren P.R. (2010). Application of a composite pain scale to objectively monitor horses with somatic and visceral pain under hospital conditions. J. Equine Vet. Sci..

[6] Gleerup K.B., Lindegaard C. (2016). Recognition and quantification of pain in horses: A tutorial review. Equine Vet. Educ..

[7] McDonnell S. (2005). Is it psychological, physical, or both?. Proc. Am. Ass Equine Pract..

[8] Van Loon J.P.A.M., van Dierendonck M.C. (2018). Objective pain assessment in horses (2014–2018). Vet. J..

[9] Lindegaard C., Thomsen M.H., Larsen S., Andersen P.H. (2010). Analgesic efficacy of intraarticular morphine in experimentally induced radiocarpal synovitis in horses. Vet. Anaesth. Analg..

[10] Van Loon J.P.A.M., van Dierendonck M.C. (2019). Pain assessment in horses after orthopaedic surgery and with orthopaedic trauma. Vet. J..

[11] Sutton G.A., Dahan R., Turner D., Paltiel O. (2013). A behaviour-based pain scale for horses with acute colic: Scale construction. Vet. J..

[12] Bussieres G., Jacques C., Lainay O., Beauchamp G., Leblond A., Cadoré J.L., Troncy E. (2008). Development of a composite orthopaedic pain scale in horses. Res. Vet. Sci..

[13] Pritchett L.C., Ulibarri C., Roberts M.C., Schneider R.K., Sellon D.C. (2003). Identification of potential physiological and behavioral indicators of postoperative pain in horses after exploratory celiotomy for colic. Appl. Anim. Behav. Sci..

[14] Costa E.D., Minero M., Lebelt D., Stucke D., Canali E., Leach M.C. (2014). Development of the Horse Grimace Scale (HGS) as a pain assessment tool in horses undergoing routine castration. PLoS ONE.

[15] Costa E.D., Stucke D., Dai F., Minero M., Leach M.C., Lebelt D. (2016). Using the horse grimace scale (HGS) to assess pain associated with acute laminitis in horses (*Equus caballus*). Animals.

[16] Van Loon J.P.A.M., Van Dierendonck M.C. (2017). Monitoring equine head-related pain with the Equine Utrecht University scale for facial assessment of pain (EQUUS-FAP). Vet. J..

[17] Coneglian M.M., Borges T.D., Weber S.H., Bertagnon H.G., Michelotto P.V. (2020). Use of the horse grimace scale to identify and quantify pain due to dental disorders in horses. Appl. Anim. Behav. Sci..

[18] van Loon J.P., Macri L. (2021). Objective Assessment of Chronic Pain in Horses Using the Horse Chronic Pain Scale (HCPS): A Scale-Construction Study. Animals.

[19] Ortolani F., Scilimati N., Gialletti R., Menchetti L., Nannarone S. (2021). Development and preliminary validation of a pain scale for ophthalmic pain in horses: The Equine Ophthalmic Pain Scale (EOPS). Vet. J..

[20] Tateo A., Maggiolino A., Padalino B., Centoducati P. (2013). Behavior of artificially suckled foals. J. Vet. Behav..

[21] Torcivia C., McDonnell S. (2021). Equine discomfort ethogram. Animals.

[22] Robertson S.A. (2012). Analgesia in foals. Compend. Contin. Educ. Pract. Vet..

[23] van Loon J.P.A.M., Verhaar N., van den Berg E., Ross S., de Grauw J. (2020). Objective Assessment of Acute Pain in Foals Using a Facial Expression-Based Pain Scale. Animals.

[24] European Commission (2010). Directive 2010/63/EU of the European Parliament and of the Council of 22 September 2010 on the protection of animals used for scientific purposes. Off. J. Eur. Union.

[25] Sherwin C.M., Christiansen S.B., Duncan I.J.H., Erhard H.W., Lay D.C., Mench J.A. (2003). Guidelines for the ethical use of animals in applied animal behaviour research. Appl. Anim. Behav. Sci..

[26] Landis J.R., Koch G.G. (1977). The measurement of observer agreement for categorical data. Biometrics.

[27] Bernard W. (2004). Colic in the foal. Equine Vet. Educ..

[28] Van Loon J.P.A.M., van Dierendonck M.C. (2015). Monitoring acute equine visceral pain with the Equine Utrecht University Scale for Composite Pain Assessment (EQUUS-COMPASS) and the Equine Utrecht University Scale for Facial Assessment of Pain (EQUUS-FAP): A scale-construction study. Vet. J..

[29] (1991). Guide for Veterinary Service and Judging of Equestrian Events.

[30] Wong D.M., Wilkins P.A. (2015). Defining the systemic inflammatory response syndrome in equine neonates. Vet. Clin. N. Am. Equine Pract..

[31] Bernard W.V., Reimer J.M. (1994). Examination of the foal. Vet. Clin. N. Am. Equine Pract..

[32] Ohmura H., Jones J.H. (2017). Changes in heart rate and heart rate variability as a function of age in Thoroughbred horses. J. Equine Vet. Sci..

[33] McAuliffe S.B., Mc Auliffe S.B., Slovis N.M. (2008). Neonatal examination, clinical procedures and nursing care. Color Atlas of Disease and Disorder of the Foal.

[34] Nagy K., Schrott A., Kabai P. (2008). Possible influence of neighbours on stereotypic behaviour in horses. Appl. Anim. Behav. Sci..

[35] Waring G. (2003). Horse Behaviour.

[36] Paradis M.R. (2006). Gastrointestinal disease. Equine Neonatal Medicine: A Case-Based Approach.

[37] Søndergaard E., Jago J. (2010). The effect of early handling of foals on their reaction to handling, humans and novelty, and the foal–mare relationship. Appl. Anim. Behav. Sci..

[38] Taffarel M.O., Luna S.P.L., de Oliveira F.A., Cardoso G.S., de Moura Alonso J., Pantoja J.C., Brondani J.T., Love E., Taylor P., White K. (2015). Refinement and partial validation of the UNESP-Botucatu multidimensional composite pain scale for assessing postoperative pain in horses. BMC Vet. Res..

[39] Da Rocha P.B., Driessen B., McDonnell S.M., Hopster K., Zarucco L., Gozalo-Marcilla M., Hopster-Iversen C., Trindade P.H.E., da Rocha T.K.G., Taffarel M.O. (2021). A critical evaluation for validation of composite and unidimensional postoperative pain scales in horses. PLoS ONE.

[40] Raekallio M., Taylor P.M., Bennett R.C. (1997). Preliminary investigations of pain and analgesia assessment in horses administered phenylbutazone or placebo after arthroscopic surgery. Vet. Surg..

[41] Price J., Catriona S., Welsh E.M., Waran N.K. (2003). Preliminary evaluation of a behaviour–based system for assessment of post–operative pain in horses following arthroscopic surgery. Vet. Anaesth. Analg..

[42] Sellon D.C., Roberts M.C., Blikslager A.T., Ulibarri C., Papich M.G. (2004). Effects of continuous rate intravenous infusion of butorphanol on physiologic and outcome variables in horses after celiotomy. J. Vet. Intern..

[43] Gleerup K.B., Forkman B., Lindegaard C., Andersen P.H. (2015). An equine pain face. Vet. Anaesth. Analg..

[44] Graubner C., Gerber V., Doherr M., Spadavecchia C. (2011). Clinical application and reliability of a post abdominal surgery pain assessment scale (PASPAS) in horses. Vet. J..

[45] Lindegaard C., Vaabengaard D., Christophersen M.T., Ekstøm C.T., Fjeldborg J. (2009). Evaluation of pain and inflammation associated with hot iron branding and microchip transponder injection in horses. Am. J. Vet. Res..

[46] McCann J.S., Heird J.C., Bell R.W., Lutherer L.O. (1988). Normal and more highly reactive horses. I. Heart rate, respiration rate and behavioral observations. Appl. Anim. Behav. Sci..

[47] Carroll J.M., Russell J.A. (1996). Do facial expressions signal specific emotions? Judging emotion from the face in context. J. Pers. Soc. Psychol..

[48] Siniscalchi M., Padalino B., Aubé L., Quaranta A. (2015). Right-nostril use during sniffing at arousing stimuli produces higher cardiac activity in jumper horses. Laterality.

[49] Corgan M.E., Grandin T., Matlock S. (2021). Evaluating the Reaction to a Complex Rotated Object in the American Quarter Horse (*Equus caballus*). Animals.

[50] Sutton G.A., Paltiel O., Soffer M., Turner D. (2013). Validation of two behaviour-based pain scales for horses with acute colic. Vet. J..

[51] Streiner D.L., Norman G.R., Cainery J. (2015). Health Measurement Scales: A Practical Guide to Their Development and Use.

[52] Duhn L.J., Medves J.M. (2004). A systematic integrative review of infant pain assessment tools. Adv. Neonatal Care.

[53] von Baeyer C.L., Spagrud L.J. (2007). Systematic review of observational (behavioral) measures of pain for children and adolescents aged 3 to 18 year. Pain.

[54] Zwakhalen S.M., Hamers J.P., Abu-Saad H.H., Berger M.B. (2006). Pain in elderly people with severe dementia: A systematic review of behavioural pain assessment tools. BMC Geriatr..

[55] Bradley C. (2001). Importance of differentiating health status from quality of life. Lancet.

[56] Mellor D.J. (2016). Updating animal welfare thinking: Moving beyond the ‘Five Freedoms’ towards ‘A Life Worth Living’. Animals.

[57] Parker R.A., Yeates J.W. (2012). Assessment of quality of life in equine patients. Equine Vet. J..

